# Comparing healthcare cost associated with the use of enzyme-inducing and non-enzyme active antiepileptic drugs in elderly patients with epilepsy in the UK: a long-term retrospective, matched cohort study

**DOI:** 10.1186/s12883-019-1587-9

**Published:** 2020-01-08

**Authors:** Simon Borghs, Laura Byram, Jane Chan, Peter Dedeken, John Logan, Victor Kiri, Matthias Noack-Rink, Philip N. Patsalos, Solène Thieffry

**Affiliations:** 1 UCB Pharma, Slough, UK; 2 UCB Pharma, Brussels, Belgium; 3 Heilig Hart Hospitaal, Lier, Belgium; 4 Stats4Pharma, Cork, Republic of Ireland; 5 FV & JK Consulting Ltd, Guildford, UK; 6 Medical Consultant, Hennef, Germany; 7 UCL Queen Square Institute of Neurology, London, UK

**Keywords:** Epilepsy, Healthcare costs, Elderly, Cytochrome P450 enzyme system, Drug-related side effects and adverse reactions, Comorbidity, Database, Hospital records

## Abstract

**Background:**

In elderly patients (≥65 years of age) with epilepsy who take medications for comorbid conditions, some antiepileptic drugs (AEDs) may alter the metabolism of other treatments and increase the risk of adverse consequences and healthcare utilisation. This analysis compares healthcare costs associated with enzyme-inducing AEDs (EIAEDs) and non-enzyme active AEDs (nEAAEDs) use in elderly patients with epilepsy.

**Methods:**

This retrospective matched cohort study used the Clinical Practice Research Datalink (CPRD) of UK primary care medical records, linked to the Hospital Episode Statistics (HES) database. Selected patients with epilepsy were ≥ 65 years and prescribed an EIAED or nEAAED between 2001 and 2010 (index) after ≥1 year without AEDs (baseline) and followed until the first occurrence of the following: end of HES data coverage, end of GP registration, or death; practice’s up-to-standard status or addition of an AED belonging to another cohort or discontinuation of the last AED of that cohort. Propensity score matching reduced confounding factor effects between cohorts. Key outcomes included time to cohort treatment failure, time to index AED treatment failure, and direct healthcare costs in 2014 Pound Sterling (£) values.

**Results:**

Overall, 1425 elderly patients were included: 964 with EIAEDs and 461 with nEAAEDs. At baseline, the EIAED cohort was older (mean age, 76.2 vs. 75.1 years) and a higher proportion were male. Baseline direct healthcare costs were similar. After matching (*n* = 210 each), and over the entire follow-up period, median monthly direct healthcare costs were higher for patients taking EIAEDs than nEAAEDs (£403 vs. £317; *p* = 0.0150, Mann-Whitney U). Costs were higher for patients remaining in the EIAED cohort after 3 follow-up years. The median time to cohort treatment failure for the EIAED cohort was 1110 days vs. 1175 days for the nEAAED cohort.

**Conclusion:**

Newly treated elderly patients with epilepsy were more likely to be prescribed EIAEDs than nEAAEDs. In matched cohorts, elderly patients with epilepsy treated with EIAEDs had higher average total direct and epilepsy-related healthcare costs than nEAAED-treated patients; this difference was greater than previously reported in the overall adult population. Changing treatment practices could improve patient care and reduce costs.

## Background

In 2017, approximately 18% of people in the United Kingdom (UK) were 65 years of age or older and this number is estimated to increase to 24% by the year 2037 [1]. A sharp upturn in the incidence of epilepsy begins in persons 60 years of age or older, largely due to stroke, traumatic brain injuries and metabolic disorders, causing this population to be the fastest growing patient segment with epilepsy [2–4]. Elderly patients with epilepsy have a high prevalence of comorbid conditions that require pharmacotherapy [5, 6]. Some currently prescribed antiepileptic drugs (AEDs), such as carbamazepine and phenytoin, induce drug-metabolising enzymes and may increase the risk of long-term adverse consequences (e.g. osteoporosis, cardiovascular risk). As a result, such AEDs have the potential to increase healthcare utilisation, including the need for concomitant medication dose escalations, more frequent primary care visits to manage other prescriptions and their side effects, and the development of comorbidities requiring additional treatment [7–9].

Compared with non-enzyme active AEDs (nEAAEDs), such as lamotrigine and levetiracetam, enzyme-inducing AEDs (e.g. carbamazepine; EIAEDs) have been associated with increased lipid levels irrespective of statin treatment in the elderly [10] and increased use of statins in newly treated patients with epilepsy [11]. In addition, a treatment switch from EIAEDs to nEAAEDs has been associated with reductions in risk markers for vascular disease, including serum lipids and C-reactive protein [12]. Despite reasons to avoid EIAED use in the elderly, a US-based study reported increased prescription of EIAEDs with increasing patient age [11].

Our previous retrospective database analysis in adult patients with epilepsy in the UK demonstrated higher long-term healthcare costs with the use of EIAEDs compared with nEAAEDs [13]. After up to 12 years of follow-up, the median monthly direct healthcare costs (in British Pound Sterling [£]) were higher for patients taking EIAEDs versus those taking nEAAEDs (£229 vs. £188, respectively). The time to treatment failure was shorter for the EIAED cohort versus the nEAAED cohort (468 days vs. 1194 days, respectively) [13].

The current analysis adds to our previous findings by comparing the long-term healthcare costs associated with the use of EIAEDs and nEAAEDs in elderly patients (≥65 years of age) with epilepsy, who are likely to have higher co-medication and comorbidity burdens. We hypothesised that because of their higher co-medication and comorbidity burdens, elderly patients would be more at risk of complications associated with EIAEDs, and therefore the cost difference between the EIAED and nEAAED cohorts would be larger than in the overall adult population.

## Methods

This was a retrospective matched cohort study comparing the long-term healthcare costs associated with the use of EIAEDs and nEAAEDs in patients ≥65 years of age with epilepsy in the UK. The methods for this analysis were similar to those applied to the full study patients with epilepsy ≥16 years of age [13]. For this analysis, patient selection was repeated based on age (≥65 years) and cohorts were newly matched.

### Patient selection and follow-up

Patients with epilepsy ≥65 years of age prescribed a new EIAED or nEAAED between 1 January 2001 and 31 December 2010 (index date) were selected from the October 2014 Clinical Practice Research Datalink (CPRD) of UK primary care medical records, linked to the Hospital Episode Statistics (HES) database (set 10). The latter contains details of all admissions, outpatient appointments and Accident & Emergency (A&E) attendances at National Health Service hospitals in England. Patients were eligible for inclusion in the study if they were ≥ 65 years of age at index date; if in the year before the index date CPRD and HES data were available; the patient received no AED prescriptions; and the patient had at least one epilepsy diagnosis at any time. At the index date, patients received a prescription for either an EIAED (carbamazepine, phenytoin, phenobarbital or primidone) or a nEAAED (gabapentin, lacosamide, lamotrigine, levetiracetam, perampanel, pregabalin, retigabine, vigabatrin or zonisamide). Selected patients were placed into one of two cohorts: those who were receiving EIAEDs or nEAAEDs. Consistent with the earlier study, the AEDs eslicarbazepine acetate, oxcarbazepine, rufinamide or topiramate were not permitted during the pre-index or follow-up period because of their mild enzyme induction profile. This approach was taken in order to avoid potential bias when comparing EIAED and EAAED cost outcomes. Patients switching between/adding within-cohort AEDs remained in follow-up. Patients were followed until end of HES data coverage, end of GP registration, or death; end of practice’s ‘up-to-standard’ status; until the addition of an AED belonging to another cohort (e.g. EIAED to nEAAED); or discontinuation of the last AED of that cohort.

### Main outcome measures

Healthcare cost outcomes were median monthly direct healthcare costs (A&E visits, AED medications, general practitioner consultations, inpatient hospitalisations, non-AED medications, outpatient non-A&E referrals) in 2014 £ values. Exposure outcomes were time to cohort treatment failure (addition of an AED belonging to a different cohort to index AED, and/or discontinuation of the last AED of the initial cohort); and time to index AED treatment failure (discontinuation of index AED or addition of *any* other AED).

### Statistical analyses

Patients from the EIAED and nEAAED cohorts were matched 1:1 based on their propensity scores derived from age, sex, general health- and cost-related characteristics, epilepsy-related variables, specific pre-index comorbidities and non-AED treatment use, and other potential confounders [13]. Propensity score matching reduces the effects of confounding for being prescribed a nEAAED versus EIAED. Outcome analyses were carried out on matched cohorts. The standardised monthly direct healthcare costs during follow-up were derived by dividing the sum of the total healthcare costs per patient by the number of months of patient follow-up. Due to the highly skewed distribution of the healthcare cost variable, differences in median healthcare costs between the two cohorts were compared using the non-parametric Mann–Whitney U test. Time to treatment failure and time to index AED failure were analysed using Kaplan–Meier analyses. Consistent with the initial study, formal power calculations for sample size were not performed for this analysis.

## Results

### Baseline characteristics

Overall, 1425 unmatched patients were selected for analysis; 964 (67.6%) patients for the EIAED cohort and 461 (32.4%) patients for the nEAAED cohort. Before matching, the EIAED cohort was older (mean age 76.2 vs. 75.1 years), included a higher proportion of male patients (51.2% vs. 41.4%), and had a higher Germaine–Smith comorbidity score (1.7 vs. 1.4) than the nEAAED cohort (Table 1). After matching, the two cohorts (*n* = 210 each) were similar on all measured confounders. In the unmatched cohort at the index date, the most commonly prescribed EIAEDs were carbamazepine (47.9%) and phenytoin (50.3%); the most commonly prescribed nEAAEDs were lamotrigine (43.6%), gabapentin (30.6%) and levetiracetam (13.0%). Although a higher percentage of patients in the unmatched nEAAED cohort were taking antihypertensive, statin, or antidepressant or antipsychotic drugs at index, a relatively large proportion of patients in the unmatched EIAED cohort had been taking these agents as well (Table 1).

**Table 1:** Baseline demographics and patient characteristics

	Unmatched population			Matched population		
	EIAED(*n* = 964)	nEAAED (*n* = 461)	*p*-value^a^	EIAED(*n* = 210)	nEAAED (*n* = 210)	*p*-value^a^
Demographics						
Age at index date, mean (SD), years	76.2 (7.4)	75.1 (7.5)	0.0076	75.7 (7.3)	74.8 (7.7)	0.2041
Age band, *n* (%)						
65–69	219 (22.7)	133 (28.9)		51 (24.3)	70 (33.3)	
70–74	217 (22.5)	115 (24.9)		54 (25.7)	43 (20.5)	
75–79	212 (22.0)	87 (18.9)		38 (18.1)	44 (21.0)	
80–84	181 (18.8)	66 (14.3)		40 (19.0)	25 (11.9)	
85–89	86 (8.9)	39 (8.5)		17 (8.1)	18 (8.6)	
≥90	49 (5.1)	21 (4.6)		10 (4.8)	10 (4.8)	
Male, *n* (%)	494 (51.2)	191 (41.4)	0.0005	105 (50.0)	99 (47.1)	0.5580
Germaine–Smith comorbidity index, mean (SD)	1.7 (2.4)	1.4 (2.2)	0.0715	1.2 (1.8)	1.2 (1.8)	0.7043
Epilepsy and treatment characteristics						
Time since first epilepsy diagnosis, mean (SD), years	3.5 (10.2)	10.9 (18.0)	< 0.0001	5.7 (13.8)	7.5 (15.3)	0.2075
Epilepsy type, *n* (%)						
Focal	154 (16.0)	70 (15.2)	0.0379	29 (13.8)	36 (17.1)	0.5268
Generalised	119 (12.3)	37 (8.0)		18 (8.6)	14 (6.7)	
Unspecified	691 (71.7)	354 (76.8)		163 (77.6)	160 (76.2)	
Index AED is first AED, *n* (%)	920 (95.4)	373 (80.9)	< 0.0001	194 (92.4)	185 (88.1)	0.1390
Most common (≥10% of patients) index AED, *n* (%)			< 0.0001			< 0.0001
Carbamazepine	462 (47.9)	0		111 (52.9)	0	
Gabapentin	0	141 (30.6)		0	52 (24.8)	
Lamotrigine	0	201 (43.6)		0	109 (51.9)	
Levetiracetam	0	60 (13.0)		0	32 (15.2)	
Phenytoin	485 (50.3)	0		94 (44.8)	0	
Pregabalin	0	59 (12.8)		0	17 (8.1)	
Most common (≥5% of patients in any category) comorbidities, *n* (%)						
Cardiovascular disease	439 (45.5)	193 (41.9)	0.1916	86 (41.0)	79 (37.6)	0.4843
Hypertension	285 (29.6)	130 (28.2)	0.5958	53 (25.2)	51 (24.3)	0.8211
Neoplasms	128 (13.3)	41 (8.9)	0.0166	17 (8.1)	18 (8.6)	0.8599
Psychiatric issues	95 (9.9)	43 (9.3)	0.7529	16 (7.6)	21 (10.0)	0.3894
Osteoporosis	30 (3.1)	27 (5.9)	0.0134	6 (2.9)	7 (3.3)	0.7781
Most common (≥5% of patients in any category) non-AED medication use, *n* (%)						
Antihypertension drugs	416 (43.2)	249 (54.0)	0.0001	102 (48.6)	99 (47.1)	0.7695
Statins	310 (32.2)	226 (49.0)	< 0.0001	86 (41.0)	91 (43.3)	0.6212
Antidepressant/antipsychotic drugs	292 (30.3)	187 (40.6)	0.0001	57 (27.1)	67 (31.9)	0.2847
Glucocorticoids	169 (17.5)	112 (24.3)	0.0027	40 (19.0)	44 (21.0)	0.6256
Sex hormones	49 (5.1)	35 (7.6)	0.0599	12 (5.7)	10 (4.8)	0.6614
Anticoagulants	46 (4.8)	52 (11.3)	< 0.0001	8 (3.8)	14 (6.7)	0.1888
Healthcare resource use, mean (SD) number						
GP consultations	46.7 (31.6)	63.7 (37.5)	< 0.0001	53.0 (31.7)	53.4 (32.6)	0.9095
A&E visits	1.1 (1.5)	0.9 (1.5)	0.1440	0.9 (1.2)	0.8 (1.3)	0.3481
Outpatient, non-A&E referrals	1.0 (1.6)	1.3 (1.5)	0.0007	1.2 (1.5)	1.2 (1.5)	0.6965
Inpatient hospitalisation	1.9 (5.3)	1.6 (2.2)	0.1005	1.4 (1.7)	1.4 (1.9)	0.6480
Hospitalisation days, mean (SD)	10.0 (18.5)	5.5 (10.4)	< 0.0001	6.2 (12.6)	6.1 (12.2)	0.9715
All direct healthcare cost in the 1-year pre-index period, 2014 £						
Median (range)	4572 (11–84,513)	4869 (274–61,048)	0.3620	4004 (11–48,356)	3814 (274–32,349)	0.8314
Epilepsy-related cost, median (range)	42 (0–24,502)	0 (0–25,822)	0.0051	42 (0–10,459)	11 (0–25,822)	0.7881

^a^Prior to t-test was used to assess differences in the means between the cohorts for continuous variables, chi-square test for categorical variables. *A&E* Accident & Emergency, *AED* Antiepileptic drug, *EIAED* Enzyme-inducing AED, *GP* General practitioner, *nEAAED* Non-enzyme active AED, *SD* Standard deviation

### Healthcare costs in the follow-up period

Over the entire follow-up period, direct all-cause and epilepsy-related healthcare costs were higher for the EIAED cohort compared with the nEAAED cohort. Median monthly direct healthcare costs were £403 for the EIAED cohort and £317 for the nEAAED cohort (*p* = 0.0150; Mann Whitney U); median epilepsy-related direct healthcare costs were £52 in the EIAED cohort and £23 in the nEAAED cohort (Table 2). With the exception of test procedures, the standardised mean and median healthcare costs were generally higher for each cost category in the EIAED cohort versus the nEAAED cohort (Table 2).

**Table 2:** Standardised monthly total, associated and epilepsy-related healthcare cost over follow-up period in the matched population

Standardised monthly costs (2014 £)		EIAED (*n* = 210)	nEAAED (*n* = 210)	*p*-value
Total all direct healthcare costs	Median (range)	403 (50, 10,856)	317 (49, 5630)	
	Mean (SD)	749 (1091)	572 (710)	0.0150^a^
A&E visits	Median (range)	1 (0, 268)	0 (0, 316)	
	Mean (SD)	9 (24)	8 (28)	0.6764
AED medications	Median (range)	16 (2, 251)	8 (2, 420)	
	Mean (SD)	32 (38)	17 (36)	< 0.0001
GP practice consultations	Median (range)	159 (12, 1222)	146 (37, 1264)	
	Mean (SD)	199 (144)	195 (162)	0.7953
Inpatient hospitalisations	Median (range)	92 (0, 10,032)	25 (0, 4852)	
	Mean (SD)	422 (1007)	275 (595)	0.0694
Non-AED medications	Median (range)	24 (0, 797)	20 (0, 551)	
	Mean (SD)	46 (74)	43 (62)	0.6954
Outpatients, non-A&E referrals	Median (range)	4 (0, 489)	3 (0, 217)	
	Mean (SD)	18 (48)	11 (23)	0.0448
Test procedures	Median (range)	11 (0, 249)	12 (0, 518)	
	Mean (SD)	23 (36)	22 (45)	0.8992
Total epilepsy-related direct healthcare costs	Median (range)	52 (2, 4124)	23 (2, 1673)	
	Mean (SD)	196 (473)	100 (196)	0.0068

^a^*p*-value based on a Mann Whitney U-test comparing distributions. All other *p*-values are based on t-test on differences in the mean costs between the cohorts. *A&E* Accident & Emergency, *AED* Antiepileptic drug, *EIAED* Enzyme-inducing AED, *GP* General practitioner, *nEAAED* Non-enzyme active AED, *SD* Standard deviation

### Direct healthcare cost by follow-up period

**Fig. 1:**
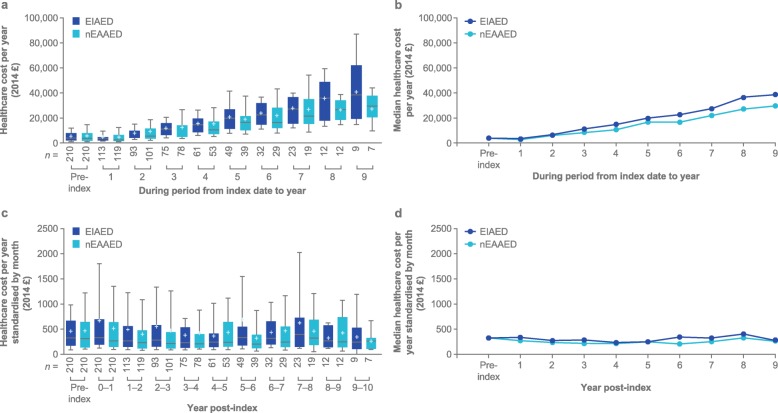
Healthcare costs for EIAED and nEAAED cohorts: **a** direct healthcare cost per year follow-up period; **b** median direct healthcare cost per year follow-up period; **c** standardised monthly cost by year post-index; **d** standardised median monthly cost by year post-index. *EIAED* enzyme-inducing antiepileptic drug, *nEAAED* non-enzyme active antiepileptic drug. For all those who started the year in question, because of a lack of available matches, data for years 10–12 are not presented. Histograms (**a** and **c**) show median (horizontal line), mean (tick mark), 25th and 75th percentile (bottom and top of box) and 10th and 90th percentile (bottom and top whisker); line graphs (**b** and **d**) show medians only

Healthcare costs for both cohorts generally increased over time, but were higher for the EIAED cohort (Fig. 1a). Starting at 3 years post-index, median direct costs per year were higher for patients prescribed EIAEDs than for patients taking nEAAEDs (Fig. 1b). For patients who completed the 7-year post-index period, mean (standard deviation [SD]) of all direct healthcare costs for the EIAED and nEAAED cohorts were £28,398 (£14,646) and £27,299 (£17,258), respectively; median direct healthcare costs for the EIAED cohort was £27,194 (*n* = 23) and £21,779 for the nEAAED cohort (*n* = 19).

### Standardised monthly cost by year post-index

In the first post-index year, median monthly direct healthcare costs were £330 in the EIAED cohort and £269 in the nEAAED cohort versus £329 and £313, respectively, during the pre-index period (Fig. 1c). Median monthly cost dropped to £269 (EIAED, *n* = 113) and £234 (nEAAED, *n* = 119) in year 2 for patients remaining in follow-up. In years 3–5, median monthly costs for both cohorts remained relatively stable, until year 6 when median standardised monthly costs for the EIAED cohort increased to £338 (*n* = 49) and then dropping to £319 in year 7 (*n* = 32) (Fig. 1d). There was stability in median monthly cost in the nEAAED cohort, reaching £245 for patients who entered year 7 (*n* = 29). Mean (SD) monthly direct healthcare costs for the EIAED and nEAAED cohorts were £698 (£1109) and £550 (£739) in year 1 and retreated toward initial pre-index cost in year 2 (EIAED: £521 [£735]; nEAAED: £437 [£568]). The mean costs then fluctuated through the remainder of the assessment period (Fig. 1c).

### Time to cohort treatment failure

**Fig. 2:**
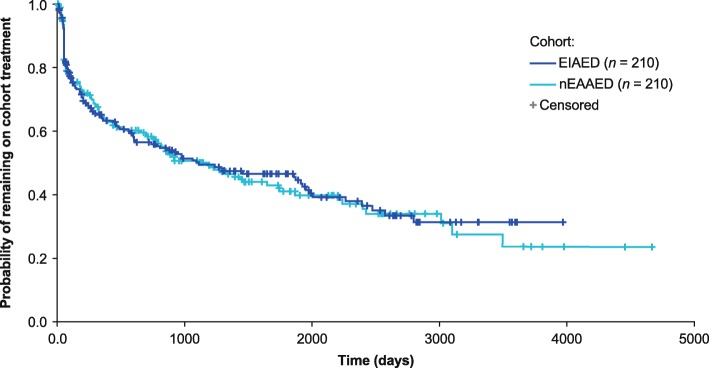
Kaplan–Meier plot of time to cohort treatment failure. *EIAED* enzyme-inducing antiepileptic drug, *nEAAED* non-enzyme active antiepileptic drug

In the matched cohorts, the median time to cohort treatment failure (allowing within-group AED switching) was 1110 days in the EIAED cohort compared with 1175 days in the nEAAED cohort (Fig. 2), with a total mean follow-up time of 806.1 versus 916.5 patient-years, respectively. The median time to the end of follow-up in the EIAED cohort was 544 days versus 659 days in the nEAAED cohort.

### Time to index AED treatment failure

**Fig. 3:**
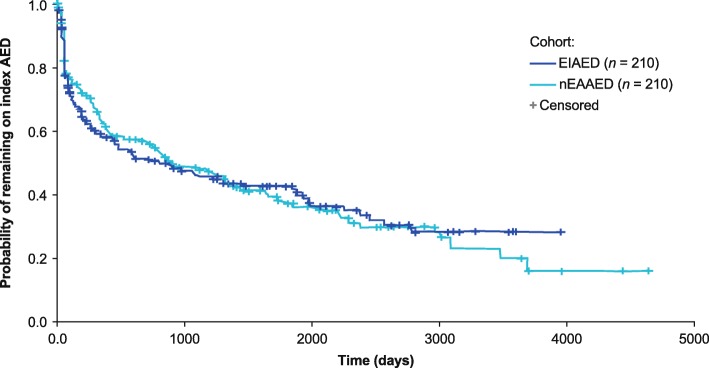
Kaplan–Meier plot of time to index AED treatment failure. *AED* antiepileptic drug, *EIAED* enzyme-inducing AED, *nEAAED* non-enzyme active AED. Patients were censored at whichever occurred first: end of HES coverage; end of data coverage; end of registration/death; end of practice’s ‘up-to-standard’ status

In the matched cohorts, the Kaplan–Meier estimated median time to index AED treatment failure was shorter for patients prescribed EIAEDs (807 days) than for those prescribed nEAAEDs (910 days) (Fig. 3). The median time to index AED treatment failure because of AED discontinuation was longer for the EIAED cohort (2467 days) than for the nEAAED cohort (1336 days).

## Discussion

We have previously reported higher long-term healthcare costs associated with the use of EIAEDs compared with nEAAEDs in adults [13]. Given a higher co-medication and comorbidity burden of the elderly compared with the overall adult population, our hypothesis was that newly treated elderly patients with epilepsy (≥65 years old) would be at greater risk of complications associated with EIAEDs, which would result in higher healthcare costs than for elderly patients treated with nEAAEDs, and that this difference would be larger than in the overall adult population.

In the UK and during the period studied, newly treated elderly patients with epilepsy were more likely to be prescribed EIAEDs than nEAAEDs, despite the known risks for drug interactions and comorbidities that EIAEDs may pose. Thus, a substantial number of older patients in the UK who initiated epilepsy therapy with an EIAED appeared to be at risk of complications associated with enzyme induction. For example, a prospective study from the Women’s Health Initiative observed that women (mean age: 63 years of age) taking AEDs had an overall higher risk of fractures than women not taking the drugs, and women taking EIAEDs had a higher fracture risk compared to those who had been prescribed nEAAEDs [14]. However, other analyses suggest that even nEAAEDs may also affect bone mineral density and increase the risk of falls and fractures in elderly patients [15]. In our study, in the unmatched patients, only 3.1% (30/964) of the patients prescribed EIAEDs and 5.9% (27/461) patients were prescribed nEAAEDs were reported to have comorbid osteoporosis. In the matched cohorts, only 2.9% (6/210) of the patients prescribed EIAEDs and 3.3% (7/210) patients were prescribed nEAAEDs had comorbid osteoporosis. Although we did not further itemize each of the overall direct costs, there exists the possibility that some of the costs were associated with fractures. Indeed as expected, the elderly population in this study did have more comorbidities than the overall adult population in our 2017 report [13]. In the matched population the baseline mean Germaine–Smith comorbidity index was 1.2 for the elderly population and 0.7 for the overall adult population. Consistent with our hypothesis, the average total direct healthcare cost over long-term follow-up was higher for elderly patients on EIAEDs compared with nEAAEDs. Also at baseline, the total median all-cause direct healthcare costs were similar between the cohorts. Direct healthcare costs included A&E visits, AED medications, GP practice consultations, inpatient hospitalisations, non-AED medications, outpatient non-A&E referrals, and test procedures. In the unmatched population at baseline, the nEAAED cohort had higher non-AED medication usage, and higher mean GP consultations and outpatient non-A&E referrals while their median epilepsy-related costs was £0. Conversely, the EIAED group had almost twice as many hospitalisation days as the nEAAED group at baseline on average. The low median epilepsy-related cost implies that few patients had epilepsy-related healthcare encounters or prescriptions; given that the study selected newly diagnosed patients, the patients were likely undiagnosed until shortly before the index date, which was the date of first AED prescription.

Total epilepsy-related healthcare costs were higher for those patients prescribed EIAEDs than for those taking nEAAEDs. Standardised median monthly direct healthcare costs were higher in the elderly cohort versus those reported in our earlier study for all patients ≥16 years of age (£403 vs. £229 for the EIAED cohorts; £317 vs. £188 for the nEAAED cohorts) [13]. The standardised monthly cost for inpatient hospitalizations, in particular, were over 3-fold higher for patients prescribed an EIAED vs. a nEAAED (£92 vs. £25), which are in stark contrast to the median inpatient hospitalisation costs reported for the overall adult population (0 for both EIAED and nEAAED) [13]. Median AED medication costs also were twice as high for the EIAED cohort vs. the nEAAED cohort £16 vs. £8; *p* < 0.0001), reflecting the greater cost associated with EIAEDs. In fact, with the exception of A&E visits, the median costs associated with each healthcare category were higher for the elderly population, particularly for those patients prescribed EIAEDs, versus those reported in the earlier study of all adults.

In matched cohorts, the time to index cohort treatment failure and index AED treatment failure were shorter for elderly patients taking EIAEDs than for those taking nEAAEDs. It should be noted that in the nEAAED cohort, however, the chance of staying within the cohort was much greater compared with the EIAED cohort because the number of AED options was higher (nine AEDs vs. three AEDs, as primidone is metabolised into phenobarbital). Of note, time to index AED treatment failure due to AED discontinuation was longer for the EIAED cohort than for the nEAAED cohort. The time to index AED treatment failure also was longer in the present analysis (807 days) compared with our earlier report (452 days) [13]. The extended time to treatment failure due to index AED discontinuation may be explained by the following: (i) traditional physician behaviour, i.e. physicians are much more familiar with carbamazepine and phenytoin as basic AEDs and are hesitant to withdraw them; (ii) medical reasons; strong EIAEDs are more complicated to withdraw than nEAAEDs; and (iii) EIAEDs are also much more difficult to combine with other AEDs, so physicians might wait longer to add another AED despite patients not being seizure free. Finally, another factor may be that elderly individuals who develop epilepsy as a consequence of stroke or vascular disease, for example, are known to have better seizure control with AED treatment compared to younger patients [16, 17].

Changing current treatment practices could potentially improve patient outcomes and reduce total direct healthcare costs in elderly patients with epilepsy in the long term. More specifically, as elderly patients are more at risk of adverse events because of EIAED use and because the difference in healthcare cost between EIAED and nEAAED use is higher in these patients compared with the overall adult population, treatment guidelines should take age more into account than they do at present. Current National Institute for Health and Care Excellence guidelines recommend carbamazepine as a first-line treatment option for adults with newly diagnosed focal seizures, and for older people with epilepsy, if carbamazepine is used, controlled-release preparations should be offered [18].

Advantages of the CPRD as a data source include it being representative of the UK population with regard to age, sex and ethnicity [19]. Furthermore, it contains over 79 million person-years of follow-up data derived from 674 primary care practices in the UK [19]. This makes it a particularly useful resource for the identification of trends that emerge over the long term. Limitations of this study include its retrospective nature, meaning that the findings may not be indicative of current clinical practice in the UK. The fact that newly treated elderly patients with epilepsy in the present study were more likely to be prescribed EIAEDs is consistent with a retrospective database analysis by Mintzer and colleagues on the use of AEDs and lipid-lowering agents. Mintzer et al. found that EIAEDs were prescribed at a higher rate with increasing age in the United States despite the potential for interactions with other medications [11]. A more recent retrospective cohort analysis on AED use by Powell et al. also used the CPRD and HES databases and showed that the use of EIAEDs in elderly patients with epilepsy had decreased from 2003 to 2016 in favour of newer nEAAEDs such as levetiracetam and lamotrigine [20]. This suggests that trends in AED use may be improving toward more appropriate options in elderly patients who are likely to be taking medications that may be affected by enzymatic induction. It also should be noted that case numbers for the longer term healthcare costs described in this study were low, so these data should be interpreted with caution. A considerable portion of unmatched patients in the nEAAED group received pregabalin or gabapentin as index AED. Pregabalin and gabapentin had both been approved for neuropathic pain as well as epilepsy during the time frame used in our analysis (years 2001–2010). We did not confirm whether the patients with epilepsy were prescribed either drug for epilepsy or another indication, such as neuropathic pain. In CPRD and HES, a direct link between prescription and diagnosis is not made. It might have been possible to infer this link if both occurred during the same visit, however, this would not have applied to all cases (e.g. those diagnosed in hospital) and thus would lack specificity. Since our study selected patients with a diagnosis of epilepsy who were untreated for at least a year, it is likely that either drug would have been prescribed for epilepsy. Finally, seizure frequency data, adverse event reporting or reasons for discontinuation could not be derived from the CPRD data; therefore, underlying reasons for the reported treatment changes are not known and cannot be put in context; this together with the sample size of the current study makes it difficult to evaluate the risk of adverse consequences previously linked to EIAED use, such as osteoporosis and vascular risk [7]. Given these limitations, however, and consistent with our earlier study [13], a strength of this analysis was the use of patient propensity score matching. Such matching reduces the effect of confounding factors and improves the accuracy of the estimated costs and time to treatment failure.

## Conclusion

This study has shown that elderly patients with epilepsy are more likely to be treated with EIAEDs than with nEAAEDs, and that elderly patients with epilepsy treated with EIAEDs had higher average total direct and epilepsy-related healthcare costs than patients treated with nEAAEDs; this difference was greater than previously reported in the overall adult population. In matched cohorts, median total direct healthcare costs were £403 per month for patients treated with EIAEDs and £317 per month for those treated with nEAAEDs. Because the elderly population with epilepsy continues to increase, it is important to identify and consider factors that may improve treatment outcomes and reduce related costs, so as to ultimately minimise the burden of epilepsy on individual patients and society as a whole.

## Data Availability

The source datasets supporting the conclusions in this manuscript are available from the National Health Service National Institute for Health Research, the Medicines & Healthcare products Regulatory Agency, and the Health and Social Care Information Centre. Our costing estimates are available by request.

## Abbreviations

A&E: Accident & Emergency
AED: Antiepileptic drug
CPRD: Clinical Practice Research Datalink
EIAED: Enzyme-inducing AED
GP: General practitioner
HES: Hospital Episode Statistics
nEAAED: Non-enzyme active AED
SD: Standard deviation
